# Basic-Clinical Analysis of Parathyroid Cancer

**DOI:** 10.3390/biomedicines13030687

**Published:** 2025-03-11

**Authors:** Lucas Fuenzalida, Sebastián Indo, Héctor R. Contreras, Daniel Rappoport, Patricio Cabané

**Affiliations:** 1 Doctorate in Medical Sciences and Clinical Specialty Program, Postgraduate School, Faculty of Medicine, University of Chile, Santiago 8320328, Chile; lucas.fuenzalida@ug.uchile.cl; 2 Department of Surgery, Clinical Hospital—University of Chile, Santiago 8320328, Chile; rappoport@uchile.cl; 3 Laboratory of Cellular and Molecular Oncology, Department of Basic and Clinical Oncology, Faculty of Medicine, University of Chile, Santiago 8320328, Chile; srindo.cofre@gmail.com (S.I.); hcontrer@uchile.cl (H.R.C.); 4 Department of Medical Technology, Faculty of Medicine, University of Chile, Santiago 8320328, Chile; 5 Center for Cancer Prevention and Control (CECAN), Santiago 8380453, Chile; 6 Department of Surgery, Faculty of Medicine, Universidad Andres Bello, Santiago 7501015, Chile; 7 Department of Head and Neck Surgery, Clinca INDISA, Santiago 7520440, Chile

**Keywords:** parathyroid cancer, primary hyperparathyroidism, biomarkers, epithelial–mesenchymal transition (EMT)

## Abstract

Parathyroid cancer (PC) presents clinically as a case of hyperparathyroidism associated with local compression symptoms. The definitive diagnosis of PC is complex as it requires unequivocal criteria of invasion in postoperative biopsy. Given the difficulty in confirming the diagnosis of PC, attempts have been made to address this problem through the search for biomarkers, mainly using immunohistochemistry. Within this theme, the phenomenon of epithelial–mesenchymal transition and cancer stem cell markers have been scarcely studied; this could eventually help discriminate between a diagnosis of parathyroid adenoma or carcinoma. On the other hand, identification of oncogenes and tumor suppressing genes, as well as epigenetic markers such as miRNAs, lncRNAs, and circRNAs all play a crucial role in tumorigenesis and have enormous potential as diagnostic tools. Furthermore, proteomic-based and inflammatory markers have also been described as diagnostic aids for this uncommon neoplasm. This review presents a clinical approach to the disease, as well as providing a state-of-the-art analysis of basic biomarkers in diagnosis and future projections in this field.

## 1. Clinical Approach

### 1.1. Primary Hyperparathyroidism

Primary hyperparathyroidism (PHPT) is a common endocrine disorder characterized by excessive and autonomous secretion of parathyroid hormone (PTH) by one or more parathyroid (PT) glands, resulting in asymptomatic or symptomatic hypercalcemia [1].

Regarding the etiology of PHPT, a spectrum of proliferative disorders in the PT gland is described: solitary parathyroid adenoma (80%), multiglandular parathyroid disease (previously known as parathyroid hyperplasia, 10–15%), multiple parathyroid adenomas (5%), and parathyroid carcinoma (~1%) [2,3].

Epidemiologically speaking, PHPT is traditionally a disease diagnosed in postmenopausal women, with a predominance in African American and Caucasian populations. Its prevalence and incidence depend mainly on the availability of PTH and serum calcium measurement, associated with screening protocols, which has favored the transition to early and asymptomatic diagnosis [4]. American studies estimate an age-adjusted prevalence of 233 and 85 per 100,000 inhabitants for female and male populations, respectively, showing a 2.5:1 female predominance and a tripling of prevalence over a 15-year period (1995–2020). In addition, with advancing age (>45 years), a pronounced increase in sex-specific incidence differences is described, with a significant female predominance [5].

Clinically, most patients are asymptomatic or present with nonspecific symptoms such as fatigue, depression, or mild cognitive impairment [6]. Persistence of the disease favors symptomatology secondary to hypercalcemia, characterized by renal involvement (nephrolithiasis and renal insufficiency), gastrointestinal disease (peptic ulcer and pancreatitis), musculoskeletal disorders (fractures on pathological bone, fibrous osteitis cystica, osteoporosis, chondrocalcinosis, and arthritis), neuropsychiatric symptoms (lethargy, confusion, psychosis, and coma), among other symptoms such as polydipsia, polyuria, abdominal pain, nausea, and vomiting [6,7]. Simultaneous hypercalcemia and elevated PTH levels (or inappropriately normal levels) represent the most important factors to diagnosticate PHPT [2]. The diagnosis of PHPT requires biochemical analysis revealing simultaneous hypercalcemia an elevated PTH levels (or inappropriately normal levels) [2].

Surgical treatment (parathyroidectomy) is the only definitive treatment for PHPT, and it is indicated in symptomatic patients or asymptomatic patients who meet the criteria determined by the Fifth International Workshop (<50 years, serum calcium > 1.0 mg/dL above the upper normal level, bone mineral density showing T Score < 2.5 in those over 50 years, fracture on pathological bone, nephrolithiasis, nephrocalcinosis estimated glomerular filtration rate (eGFR) <60 mL/min, or hypercalciuria) [8,9]. Classically, surgical management of PHPT involves bilateral cervical exploration; however, advances such as intraoperative PTH measurement and improved preoperative localization methods have favored the implementation of minimally invasive techniques, remote approaches (retroauricular, axillary, submammary, transoral), and robotic-assisted surgery. Thus, parathyroidectomy is an active area of research, where various modern techniques claim better aesthetic outcomes, surgical times, or postoperative recovery [7].

### 1.2. Parathyroid Cancer

As previously mentioned, parathyroid carcinoma (PC) is the least common etiology in the context of PHPT (about 1%). It is an uncommon endocrine neoplasm, currently accounting for 0.005% of malignant neoplasms in the western population [10]. The estimated annual incidence of this condition is 3.5–5.7 cases per 10,000,000 individuals, with a peak incidence in the fifth decade and no gender differences. A recent retrospective study of the SEER database analyzed 609 cases of PC, determining a higher incidence in males (52.2%), predominance in Caucasian population (75.4%), average age at diagnosis of 62 years, mainly adenocarcinoma histology (99.7%), with 36.8% of cases being organ-confined at the time of diagnosis. Risk factors for mortality include older age (>40 years), tumor size >4 cm, male gender, metastasis, and poor histological differentiation [11].

The etiology of PC is unknown, but an increased incidence has been associated with cervical irradiation and stage V chronic kidney disease [12]. This cancer typically presents as spontaneous disease; however, it can also be associated with specific syndromes such as hyperparathyroidism-jaw tumor syndrome (HPT-JT) or multiple endocrine neoplasia (MEN) types 1 and 2A. Genetically, a relationship has been established between mutations in the CDC73 gene (which codes for parafibromin) and spontaneous PC (present in up to 70% of cases) and familial forms [10,12]. Parafibromin is a tumor suppressor protein that can be detected by immunohistochemistry, and its decrease is associated with increased cell proliferation, apoptosis, and chromosomal instability [13].

PC presents clinically as a case of hyperparathyroidism (described earlier) which precedes local compression symptoms. Additionally, PC tends to have higher hormonal activity than PT adenomas and hyperplasias, resulting in serum PTH levels up to 10 times the normal value and hypercalcemic crisis in up to 12% of patients (serum calcium > 14 mg/dL, oligoanuria, and neuropsychiatric symptoms). Among the symptoms associated with PHPT, renal (nephrolithiasis and renal insufficiency) and skeletal (bone pain, fractures in pathological bone, osteoporosis, etc.), manifestations are most common. On physical examination, a palpable cervical mass can be found in 40–70% of cases, which may be associated with compressive symptoms of the upper airway and digestive tract. It is described that up to 10% of these carcinomas are non-functioning, and in consequence are diagnosed later, usually due to compressive symptoms. Cervical lymph nodes are the main site of metastasis, present in 15–20% of cases, followed by distant metastasis to lung, liver, and bone (1%) [14,15].

Clinical suspicion of PC is important due to the similarity with other proliferative pathologies within the PHPT spectrum and the rarity of this carcinoma. In this context, it is important to remember the preoperative suspicion criteria: male gender, young age (<50 years), concomitant renal and skeletal manifestations, palpable cervical mass, paralysis of the laryngeal nerves, plasma PTH levels > 10 times the normal level, serum calcium > 14 mg/dL or hypercalcemic crisis, local invasion of adjacent structures and suspicion of metastasis on imaging studies [10,14].

Despite the previously described criteria, the definitive diagnosis of PC is complex as it requires unequivocal criteria of invasion in postoperative biopsy, including angioinvasion, lymphoinvasion, perineural invasion, invasion of neighboring soft tissues and musculoskeletal structures, thyroid, esophagus, etc., and/or presence of lymph node or distant metastasis. The aforementioned are absolute criteria, and meeting just one of them is sufficient for diagnosing PC. The cytological or histological characteristics of the tumor are not sufficient to make the diagnosis. This determines the difficulty in discriminating between PT adenoma and carcinoma. In Table 1, you will find a comparison between PT adenoma and PC. Consequently, malignancy-associated criteria have been established. The presence of these criteria indicates a high risk of malignant behavior, acquiring the name of “atypical parathyroid tumor” (however, not sufficient for PC diagnosis). These criteria include trabecular or “sheet-like” architecture, wide bands of intraglandular fibrosis, diffuse cellular atypia, enlarged nucleoli, >5 mitoses per 10 mm^3^, atypical mitoses, coagulation necrosis, and capsular involvement without extension into the periglandular fibroadipose tissue [3,16].

Given the difficulty in confirming the diagnosis of PC, attempts have been made to address this problem through the search for biomarkers using immunohistochemistry (IHC); but none have proven to be pathognomonic of this disease (see Section 2) [16].

Within this theme, the phenomenon of epithelial–mesenchymal transition (EMT) and cancer stem cells (CSC) markers have been scarcely studied; this could eventually help discriminate between a diagnosis of parathyroid adenoma or carcinoma.

Regarding treatment, surgical management is the only curative option, prior to medical management of metabolic abnormalities. This consists of an en bloc resection of the primary tumor, along with the ipsilateral thyroid lobe and isthmus, as well as involved pre-thyroid muscles and central neck lymph node dissection [10,14].

On the other hand, PC is considered radio-resistant; however, some series report lower disease progression and possibly lower recurrence when used as postoperative adjuvant treatment [12]. Despite this, the use of adjuvant radiotherapy has not shown benefits in overall survival. Additionally, a better 5-year survival is described in patients treated surgically compared to isolated radiotherapy [11]. Currently, its adjuvant use is reserved for patients at high risk of locoregional recurrence [17,18].

Similarly, the use of chemotherapy in PC has historically shown low to no efficacy. A recent report describes a favorable response with the use of the multikinase inhibitor Sorafenib in a case of PC with lung metastases, although further evaluation is needed [10,19].

Reports by Christakis et al. highlight that there have been no significant changes in the management and prognosis in overall survival in the past 35 years at the University of Texas MD Anderson Cancer Center, underscoring the importance of promoting research and progress in treatments for this cancer [17].

Long-term survival is favorable, with 5- and 10-year survival rates ranging from 85–91% and 49–87.6%, respectively. In general, mortality is secondary to complications associated with hypercalcemia, rather than tumor burden [12].

## 2. State-of-the-Art Analysis in Parathyroid Cancer: Diagnosis and Utility of Frequent Biomarkers

### 2.1. Diagnosis and Serum Biomarkers

As previously mentioned, the definitive diagnosis of PC requires unequivocal criteria for invasion in the postoperative biopsy (angioinvasion, lymphoinvasion, perineural invasion, invasion of neighboring soft tissues and musculoskeletal structures, thyroid, esophagus, etc., and/or presence of lymph node or distant metastasis). The mentioned criteria are known as absolute criteria, and meeting just one is sufficient for the diagnosis of PC. On the other hand, the cytological or histological characteristics of the tumor are not sufficient to make the diagnosis, which determines the difficulty in discriminating between PT adenoma and carcinoma. Criteria associated with malignancy can be reviewed in the Introduction section [3,16]. Given the difficulty in confirming the diagnosis of PC, attempts have been made to address this problem by searching for biomarkers mainly through IHC; however, none have been found to be pathognomonic of the disease [16].

In conclusion, due to the low frequency of this cancer, most biomarker studies in PC are in early phases, do not have clear definitions regarding results, show variations in populations used and reporting methods, and also have a small sample size (1–57 cases per study), which has made it difficult to perform statistical analyses. In this context, a recent systematic review of potential biomarkers in PC by Davies et al. significantly identified (in at least one study) 5 markers in serum and 13 in tissue. Specifically, the markers that showed the strongest evidence in serum are calcium and PTH (15 and 16 studies, respectively), with a cutoff of 3 mmol/L (12.02 mg/dL) and >3 times the upper limit of normal (ULN) for calcium and PTH, respectively, in order to suspect PC. In addition, the presence of elevated alkaline phosphatase or creatinine, as well as decreased 25-hydroxyvitamin D, are independently associated with suspicion of PC. It is likely that these latter markers are associated with a biochemical manifestation of disease severity rather than directly related to the pathophysiology of the disease (as is the case with serum calcium and PTH) [20].

### 2.2. Frequent Tissue Biomarkers

Multiple biomarkers have been described in tissue (Table 2), which implies access to tissue and post-surgical analysis by the pathologist. Despite the existence of several markers, none of them are pathognomonic of PC and it is currently an active area of research, where every study is an advancement.

Parafibromin is the main biomarker studied in tissues; it is a tumor suppressor protein, encoded by the Cell Division Cycle 73 (CDC73, previously HRPT2) gene. Its function lies in the repression of cyclin D1, consequently stopping the cell cycle, and it is also associated with the regulation of the P53 pathway. Mutations in this gene are associated with decreased function and detection by IHC [16]. As a result, the absence of parafibromin is associated with diagnostic evidence and worse prognosis in PC. Over 19 scientific articles have described its association with PC, showing significant results in 7 of them [20]. Despite the above, there is an overlap in the presence of this gene and protein between PC and adenomas, as CDC73 mutations have been described in 1–6% of adenomas. Given the low frequency of this cancer, a nearly perfect specificity is required for its use as a screening tool in PT tumors (as a standalone marker), which complicates its use in this aspect. However, it is a strong tool for ruling out malignancy in case of positive immunoreactivity. It should be used while considering the clinical context of the patient, and currently it is associated with other markers [21].

Galectin-3 is a glycoprotein involved in pathophysiological processes such as fibrosis, inflammation, and cancer. It plays a regulatory role in the tumor microenvironment, specifically by suppressing T-cells through inhibition of T-cell receptor (TCR)-mediated signaling. Its overexpression has been associated with tumor growth and invasion [22]. Six scientific articles have associated the overexpression of galectin-3 with PC, but there are reports that raise doubts about the utility of this marker [20]. Recently, Mohammed et al. reported only 15% positivity in PC, showing a poor profile as a biomarker: sensitivity (Sen) 6%, specificity (Spe) 29%, positive predictive value (PPV) 19%, and negative predictive value (NPV) 10% [23]. Further studies are needed for this biomarker.

Ki67 is a nuclear protein expressed in proliferating cells (active phase of the cell cycle) and downregulated during the G0 phase. Therefore, increased staining for this antigen suggests a higher proportion of proliferative cells in the histological sample. It is a diagnostic and prognostic tool used in multiple types of carcinomas (especially colorectal), where staining >5% may aid in the diagnosis of PC, although it does not yet qualify as a diagnostic criterion (thus, it is used in association with other markers). Nonetheless, there are reports that do not show significant differences between adenomas and PC, indicating the need for further studies [16,20,24].

PGP9.5 (protein gene product 9.5) is the protein product of UCHL1 (ubiquitin carboxyl-terminal esterase L1) and is mainly expressed in nervous and neuroendocrine tissues. Its presence has been associated with aggressive neoplasms (colorectal, prostate, gastric, and pulmonary). There are at least two studies where PGP9.5 is reported to be overexpressed in PC, but further studies are needed to validate these findings [20,25].

APC (*Adenomatous polyposis coli*) is a tumor suppressor gene that acts by inhibiting the Wnt signaling pathway. It can be evaluated through polymerase chain reaction (PCR) or its protein product via IHC, and its loss of expression has been associated with carcinogenesis. Currently, it is also linked to other carcinomas such as colon and liver cancer. At least two studies have shown a significant association with PC, but, similar to other markers, there are articles that have not demonstrated this association, emphasizing the need for further studies on this topic [20].

The protein p27 acts as a tumor suppressor and a cyclin-dependent kinase inhibitor, which slows down the cell cycle. Loss of p27 expression is suggestive of PC, with at least one statistically relevant study supporting this association [16,20].

The decreased expression of the calcium-sensing receptor (CaSR) has been reported in PC, which is a rare phenomenon in benign PT tumors. However, the reports on this association are inconsistent, which makes it challenging to use CaSR as a reliable biomarker. Filamin A is a scaffold protein that binds to the CaSR and facilitates the activation of the MAPK pathway. In a study by Storvall et al., cytoplasmic expression of Filamin A was significantly higher in PC compared to adenomas, suggesting its potential as a biomarker for PC [16,26].

**Table 2:** Frequently used markers studied in PC and main characteristics.

Marker	Source	Characteristics	References
**Calcium**	Serum	**Increased** >3 mmol/L (12.02 mg/dL) is suspicious of PC. >3.49 mmol/L (14 mg/dL) is highly suggestive of PC. Associated to hypercalcemic crisis. Directly related to disease pathophysiology.	[27,28]
**PTH**	Serum	**Increased** >3× upper normal level is suspicious of PC. >10× upper normal level is highly suggestive of PC. Directly related to disease pathophysiology.	[27,28,29,30]
**Alkaline phosphatase**	Serum	**Increased** Significantly associated with PC, however it corresponds to a biochemical manifestation of the disease. Reflects severity, not a direct indicator of PC.	[27,28,31,32]
**25-hydroxy vitamin D**	Serum	**Decreased** Significantly associated with PC, however it corresponds to a biochemical manifestation of the disease. Reflects severity, not a direct indicator of PC.	[33]
**Parafibromin**	Tissue	**Mutated/decreased** Tumor suppressor protein encoded by CDC73 gene. Function: repression of cyclin D1 (cell cycle halt). Detectable by IHQ. 1–6% overlap in mutations with PT adenomas.	[23,33,34,35,36,37]
**Galectin-3**	Tissue	**Increased** Glycoprotein associated with tumor growth and invasion. Regulatory role in tumor microenvironment (T-cell suppression). Overexpressed in PC, however poor profile as standalone biomarker.	[23,37,38,39,40]
**Ki67**	Tissue	**Increased** Nuclear protein expressed in proliferating cells and downregulated in G0 phase. Commonly used in colorectal cancer. Diagnostic aid if >5%.	[23,30,33,37,40]
**PGP 9.5**	Tissue	**Increased** Protein product of UCHL1 (deubiquitinating enzymes). Associated with aggressive neoplasms (colorectal, prostate, gastric, and pulmonary). Further studies are needed.	[37,41]
**APC**	Tissue	**Decreased** Tumor suppressor gene that acts by inhibiting the Wnt signaling pathway. Loss of expression is associated to carcinogenesis (mainly colon and liver). Further studies are needed.	[23,42]
**P27**	Tissue	**Decreased** Tumor suppressor protein and a cyclin-dependent kinase inhibitor which slows down the cell cycle (G1 arrest). Associated with cellular differentiation, motility, migration, and apoptosis.	[43,44]
**CaSR**	Tissue	**Decreased** Diminished expression of calcium-sensing receptor reported in PC, which is a rare phenomenon in benign PT tumors. Reports are inconsistent, further studies are needed.	[16,26]

### 2.3. Infrequent Biomarkers

Apart from the markers mentioned above, there are limited studies on other markers such as AgNOR, CDC73, cyclin D1, P21, P53, Rb (retinoblastoma gene), BCL2, BAX, Mdm2, and PTEN, among others. Most of these are individual studies and often lack statistical analysis. Undoubtedly, this is an area of research that is still not well developed but holds great promise for the future. It is important to note that no single biomarker has been able to differentiate PC from adenomas on its own, but rather these markers can serve as auxiliary tools in diagnosis and prognosis [3,20].

## 3. Epithelial–Mesenchymal Transition and Cancer Stem Cells in Parathyroid Carcinoma

### 3.1. Defintions and Main Characteristics

The presence of CSC and patterns of dedifferentiation, such as EMT, have been established in various types of cancer. Specifically, EMT is a cellular process in which an epithelial cell acquires a mesenchymal phenotype and behavior due to the downregulation of specific epithelial markers (such as cytokeratin and E-cadherin) and upregulation of mesenchymal markers (such as fibronectin, N-cadherin, and vimentin). Consequently, these cells acquire a fibroblast-like morphology, lose apico-basal polarity, as well as interaction with the basement membrane and therefore exhibit increased migratory capacity and invasive properties [45]. This process is regulated by complex molecular pathways and can be described based on certain molecular markers. The transcription factors (EMT-TFs) involved in this process include Zeb1, Zeb2, Snail1, Snail2 (Slug), and Twist1, which are responsible for silencing genes expressed in epithelial cells and inducing those specific to mesenchymal cells [46].

At the post-translational level, increased expression of certain proteins such as N-cadherin, vimentin, fibronectin, and matrix metalloproteinases (-2, -3, -9) and downregulation of E-cadherin, cytokeratin, occludins, and desmoplakin, among others, are canonical events in this cellular process [47].

Cancer stem cells (CSCs) or tumor-initiating cells are cancer cells that share characteristics with stem or progenitor cells. Of particular note is their ability to differentiate into other cell lineages and undergo self-renewal through symmetric (generating two stem cells) or asymmetric (generating one stem cell and one differentiated cell) cell division. CSCs are involved in tumor formation, invasion, metastasis and even resistance to chemotherapy and radiotherapy. There are multiple methods for detecting CSCs, with the characterization of CSCs using cellular biomarkers being a prominent approach. These biomarkers typically correspond to membrane antigens and transcription factors and may vary depending on whether the tumors are solid or hematological cancers. In solid tumors, prominent surface markers include CD24, CD44, CD90, CD133, and EpCAM, while intracellular markers such as Sox2, Oct-3/4, and Nanog are also used, among others [48,49].

### 3.2. Epithelial–Mesenchymal Transition

There are limited reports specifically aimed at evaluating these phenomena in PC. According to a recent review by Uljanovs et al., the low incidence of PC has hindered studies on epithelial–mesenchymal transition in this cancer [16].

Fendrich et al. compared the expression of EMT markers (E-cadherin, Snail, and Twist) using IHC in normal PT tissue (2 cases) and PT adenomas (25 cases), hyperplasia (25 cases), and PC (9 cases). In all cases of PC, loss of membranous staining of E-cadherin was observed, showing a characteristic cytoplasmic staining pattern indicative of EMT; while membranous staining was preserved in normal tissues and benign proliferative disorders. On the other hand, Snail and Twist lost the homogeneous expression pattern seen in the control groups, being limited to the invasive front in the cancerous tissue samples. These changes in marker expression observed in PC suggest an important role of EMT in the tumorigenesis of this cancer. Although further studies are needed, this could be a potential tool to distinguish between PT adenomas and carcinomas [50].

Schneider et al. further supported the previously described change in immunostaining pattern and compared the expression of E-cadherin in atypical PT adenomas (currently known as atypical parathyroid tumors), benign PT adenomas, and PC. It was concluded that atypical tumors showed membranous staining of E-cadherin, characteristic of benign proliferative disorders of PT tissue, in contrast to PC. The potential use of E-cadherin as a differentiating marker between these two clinical entities was suggested [51].

Meanwhile, Pandya et al. conducted a genomic profiling of 17 cases of PC using whole-exome sequencing. Among the results, recurrent somatic mutations were described in the Zeb1 gene (3 cases), a transcriptional repressor associated with tumor invasion and metastasis through the activation of EMT [52].

In addition to the previously mentioned biomarkers, the study of intermediate filaments has shown that cytokeratin 19 presents a progressive upregulation in proliferative PT lesions (adenoma, hyperplasia, and cancer), statistically significant but markedly heterogeneous. Further studies of this intermediate filament in PC are recommended. Separately, vimentin corresponds to a mesenchymal intermediate filament with important functions in cell mobility, signaling, and migration; however, it is difficult to study in PT tissue due to its glandular histology. Recently, Ulijanovs et al. described that the fraction of parenchymal cells positive for vimentin varies in healthy tissue, hyperplasia, adenoma, and carcinoma, with percentages of 9%, 11.7%, 19.3%, and 36.8%, respectively. Regarding location, in healthy tissue, vimentin expression is only perinuclear, while carcinoma shows significant cytoplasmic expression, and adenomas and hyperplasias show a combination of both patterns [16,53].

### 3.3. Cancer Stem Cells

CD44, a surface glycoprotein and a recognized CSC marker, has been less studied in PT pathology, and currently, is not believed to play a significant role in PT tumors given its absence in normal PT tissue and proliferative disorders. This is interesting considering that, generally, neuroendocrine tumors express this marker. This discrepancy could possibly be explained by the fact that PT tissue has an embryological origin in the neural crest, which is CD44(−), whereas neuroendocrine tumors that are CD44(+) originate from the endoderm. This could also explain the presence of E-cadherin, Snail, and Twist in normal and benign proliferative PT tissue. Based on this, it would be interesting to evaluate markers derived from the neural crest that may be useful in PC. Additionally, CD56, a membrane glycoprotein and a member of the immunoglobulin superfamily, has been described as a negative marker in PC, despite being commonly seen in neuroendocrine tumors of other organs. CD56 is not expressed in normal PT tissue or proliferative disorders, and its presence can help rule out a PT origin of the tumor [16,53].

**Figure 1:**
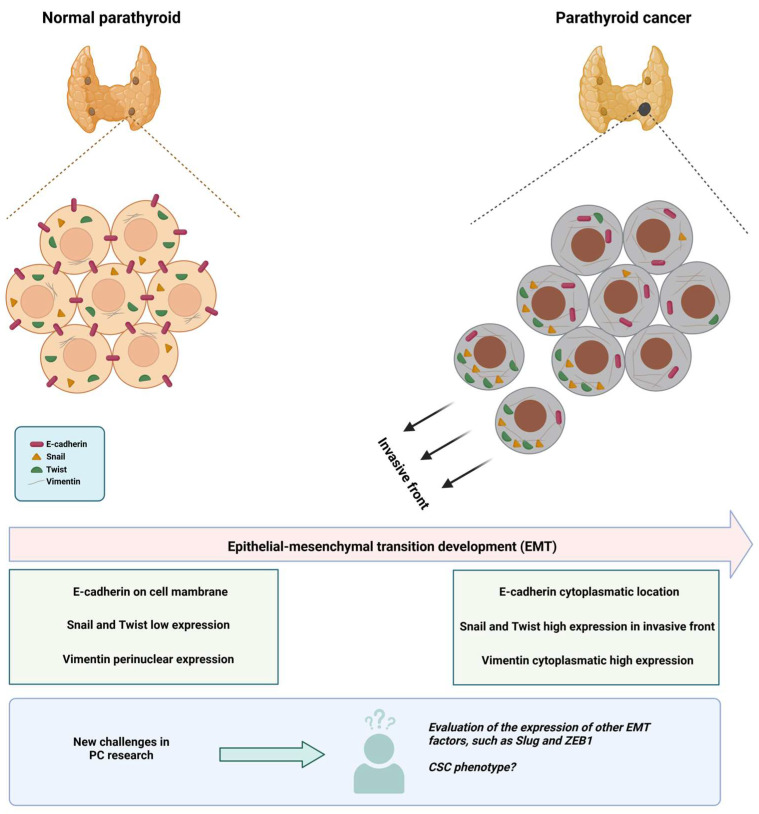
**EMT and new challenges in CP research.** The figure shows the changes that have been determined to date in normal parathyroid tissue vs. PC, emphasizing the EMT process. In the lower part of the figure, we highlight that the EMT process has not been completely elucidated, with the determination of other factors that participate in the process (such as Slug and ZEB1) still missing; also the evaluation of the CSC phenotype is still very incipient in this cancer. The figure was constructed based on the results obtained by Fendrich et al. [50], Schneider et al. [51], and Uljanovs et al. [53].

Taking all this together, we propose that studying EMT and the CSC phenotype in parathyroid cancer is an area to be explored and with great research opportunities, such as describing what happens with the Slug and Zeb1 factors (determining expression levels and subcellular localization), and how the EMT process could be stimulating the generation of CSCs (Figure 1). The evaluation of these characteristics could establish new therapeutic targets and provide CP patients with a more accurate prognosis.

## 4. Parathyroid Carcinoma Genomics

Advances in genomics are emerging and have enormous potential as they could be used relatively simply and reliably as diagnostic tools or decision aids.

Firstly, the CDC73 gene (also known as HRPT2) is a tumor suppressor gene that encodes a protein called parafibromin (whose function has been previously mentioned). Multiple somatic and germline mutations associated with partial or total inactivation of this gene have been identified. Somatic mutations in this gene are estimated to be the main genetic alteration in sporadic PC, present in two-thirds of cases (reports vary between 9 and 70%). Germline mutations are associated with HPT-JT syndrome, a population with a 20% probability of developing PC [54,55].

Secondly, the PRUNE2 gene encodes the homonymous protein, which has tumor suppressor activity by suppressing the activity of RhoA, inhibiting oncogenic transformation. Recently, Yu et al. detected several mutations via whole-exome sequencing in 18% of patients with PC but did not describe this mutation in PT adenomas. This could represent PRUNE2 as a marker that differentiates between both conditions [55,56].

Furthermore, the CCND1 gene acts as an oncogene in PT adenomas through the PTH-CCND1 rearrangement. This gene encodes cyclin D1, whose amplification has been observed in up to 71–90% of PC and is associated with cell proliferation. Recent studies have shown that amplification of CCND1 can be mutually exclusive with somatic mutations in CDC73, suggesting alternative mechanisms of malignancy [52,55].

On the other hand, multiple endocrine neoplasia type 1 (MEN1) is an autosomal dominant endocrine disorder caused by mutations in the MEN1 gene. While this syndrome is commonly associated with PT adenomas or hyperplasia, around 1% of cases may present with PC. Recent studies indicate that mutations in this gene may be present in approximately 13 to 31% of cases of PC, so additional studies could position it as a marker of the disease [55].

Genes associated with the PI3K/AKT/mTOR and Wnt signaling pathways have also been implicated in PC. The first signaling pathway plays a physiological role in cell growth and survival, and its overactivation has been associated with various types of cancers, particularly through increased cell growth, angiogenesis, suppression of senescence and autophagy, as well as increased metastatic potential. Mutations in the PI3K/AKT/mTOR pathway have been described in 13–20% of PC cases, making it a potential target for inhibitors of this pathway [55,57].

Similarly, the Wnt signaling pathway is physiologically associated with cell proliferation, survival, and apoptosis. Aberrant activation of this pathway has also been implicated in multiple neoplasms. It has been described that CDC73 regulates this pathway through stabilization of beta-catenin. In addition, recent studies have identified a regulatory role of this pathway through genetic and epigenetic changes in APC and RNF43, observed in rare cases of PC as well as colorectal and endometrial carcinomas [55].

Finally, somatic mutations have been described in other genes such as TERT, which is associated with telomerase activity through the coding of its catalytic subunit. Additionally, mutations have been identified in genes AKAP9 (signal transduction pathway), FAT3 (cellular adhesion), and ZEB1 (EMT), the latter of which was mentioned previously [55].

## 5. Epigenetics: miRNAs, lncRNAs, circRNAs

Epigenetics plays a crucial role in tumorigenesis. It encompasses all intracellular mechanisms involved in the modulation of gene expression without altering the DNA sequence. Within this field, the action of non-coding RNAs (ncRNAs) at the post-transcriptional level is noteworthy. These ncRNAs can be classified based on length into small RNAs (sRNAs) or long non-coding RNAs (lncRNAs), with lengths less than or greater than 200 nucleotides, respectively. Specifically, the former group includes microRNAs (miRNAs), while the latter encompasses a circular group of lncRNAs called circRNAs. Similar to tissue biomarkers, ncRNAs have been used to differentiate adenomas from carcinomas, with variable results, and so far, no pathognomonic marker for the disease has been identified [58].

### 5.1. miRNAs

MiRNAs (or miRs) regulate gene expression at the post-transcriptional level, usually through negative regulation of mRNA, specifically by binding to complementary sites (typically in the UTR region). These miRNAs have different expression patterns in tissues and serum, with the latter location providing the theoretical basis for liquid biopsies, with potential applications in non-invasive diagnosis and monitoring. Specifically, in the context of the PT gland, miRNAs participate in the regulation of hormonal synthesis and secretion, as well as tumorigenesis [59].

At the serum level, Wang et al. described a significant upregulation of MiR-27a-5p in serum exosomes of patients with PC, revealing an area under the curve (AUC) of 0.859. It is relevant that this specific miRNA participates in the activation of the Wnt/β-catenin signaling pathway, playing a key role in the process of EMT [60].

Also, Krupinova et al. reported that miR-342-3p is significantly decreased in PC, with an AUC of 0.89 when used as a biomarker [59,61].

Studies evaluating miRNA expression at the tissue level have shown variable results. Corbetta et al. found that miR-296 (downregulated in PC), miR-222, and miR-503 (upregulated in PC) varied significantly between adenomas and PC. On the other hand, higher levels of HGS mRNA (hepatocyte growth factor receptor-regulated tyrosine kinase substrate) were observed in PC, which has been associated with EMT-related phenomena such as downregulation of E-cadherin and subsequent increase in invasion and metastasis. Interestingly, HGS mRNA is a direct target of miR-296, and miR-296 undergoes significant downregulation during tumor progression. Consequently, an indirect role of miR-296 in the carcinogenesis of PT is suggested [62].

In parallel, Rahbari et al. reported significant variation of three miRNAs in PC: miR-26b, miR-30b, and miR-126. Notably, miR-126 showed better characteristics as a biomarker, with an AUC of 0.776 [63].

Vaira et al. described that aberrations in the expression of miRNAs from the C19MC cluster are a characteristic of PC compared to PT adenomas. This cluster of miRNAs is usually silenced by hypermethylation in adults, and its association with various human tumors has been described, promoting tumor invasion and metastasis. Specifically, within this cluster, miR-517c showed the most notable difference, correlating with tumor weight, serum calcium, and PTH levels [64].

Additionally, increased expression of miRNAs from the C19MC cluster and miR-372 has been identified in PC distant metastases [59]. Finally, Hu et al. report that in a Chinese population (which has a higher incidence of PC, accounting for 5–7% of HPTP cases), the following miRNAs showed differential expression between PC and PT adenomas: miR-139, miR-222, miR-30b, miR-517c, and miR-126. Specifically, the combination of miR-139 and miR-30b was found to be the best biomarker with an AUC of 0.89 [59,65]. It is important to highlight that most of these studies are limited by small sample sizes due to the low frequency of this cancer.

### 5.2. lncRNAs and circRNAs

LncRNAs and circRNAs primarily act as endogenous competitive RNAs (binding to miRNAs and inhibiting their function), while circRNAs can also be translated into functional micropeptides. There are limited studies on these ncRNAs in PC. In brief, Jiang et al. described that a profile composed of the lncRNAs: LINC00959, lnc-FLT3-2:2, lnc-FEZF2-9:2, and lnc-RP11-1035H13.3.1-2:1 had an AUC of 0.88, with Sen: 81.8% and Spe: 83.9% for differentiating adenomas from PC [66].

Furthermore, Zhang et al. recently described those alterations in the expression of lncRNAs PVT1 and GLIS2-AS1 had AUC values of 0.871 and 0.860, respectively [67]. Additionally, Hu et al. determined that circRNA_0075005 had an AUC of 0.77 for the same function [58,65].

## 6. Proteomics

There are limited studies on proteomic-based biomarkers in PC. Ciregia et al. documented 33 differentially expressed proteins between PC and PT adenomas. Among these, UCH-L1 (or PGP9.5) is notably overexpressed. It is an enzyme that belongs to the deubiquitinase family and is associated with carcinogenesis pathways, cell growth, DNA repair, and apoptosis. Additionally, a significant increase in ANXA2 protein has been described; a marker of aggressiveness in multiple gastrointestinal cancers, which acts by calcium-mediated binding to membrane phospholipids. Other potential markers could be MDH1, CLIC1, and SOD2 [55,68].

## 7. Inflammatory Markers

In addition to the previously described markers, the use of preoperative inflammatory markers to distinguish PC from PT adenomas is noteworthy. For example, Ohkuwa et al. evaluated the neutrophil–lymphocyte ratio, platelet–lymphocyte ratio, and lymphocyte-monocyte ratio (LMR), and found that LMR is significantly decreased in PC and is an independent predictor of this cancer when <4.85 [69]. Table 3 presents a summary of genomic, epigenetic, proteomic and inflammatory biomarkers mentioned previously.

## 8. Use of Panels

Given that none of the mentioned markers are pathognomonic of parathyroid cancer, the creation of panels, nomograms, or other systems that allow for the combination of different markers has become common. These instruments offer an increased sensitivity, specificity, positive predictive value, and negative predictive value therefore maximizing the diagnostic performance of these markers.

Briefly, we present some examples: Kumari et al. described that a panel combination of parafibromin, galectin-3, and PGP9.5 presents a Sen: 50%, Spe: 97.9%, and a predictive accuracy of 95.4% [37]. On the other hand, Truran et al. determined that the use of an immunohistochemical panel using the same markers described previously plus Ki67 is better than the isolated use of any single marker (Sen: 79% and Spe: 100%). These authors point out that a positivity greater than 5% of the cells to Ki57 is considered suspicious of being PC [25]. Finally, Silva-Figueroa et al. developed a nomogram using IHC biomarkers: parafibromin, Rb, Ki67, E-cadherin, and galectin-3, which showed an AUC of 84.9% (adjusted AUC for optimism: 80.5%) [70].

## 9. Conclusions

The progress made in the field of PC in recent years is evident; nevertheless, it remains a poorly studied cancer. Current limitations lie in the extremely low incidence presented by this neoplasm, therefore, making prospective studies a challenge. In this regard, the identification of new diagnostic markers should be approached via simple methodologies, such as liquid biopsy in patient serum or immunohistochemistry staining in paraffin-embedded biopsies. However, the lack of fresh tissue samples should be compensated with the use of cellular cultures, highlighting the current use of patient-derived organoids (3D cultures). The aforementioned could aid in medical and surgical decision-making. Therefore, it is crucial to establish collaborative basic-clinical research projects that can identify new markers and favor a more efficient diagnostic process, as well as help guide therapeutic and follow-up strategies.

## Figures and Tables

**Table 1:** Comparative table for parathyroid adenoma and PC.

	Parathyroid Adenoma	Parathyroid Cancer
**Definition**	A benign neoplasm of a parathyroid gland resulting in autonomous secretion of PTH.	A malignant neoplasm originating from parathyroid tissue, characterized by invasive growth and potential metastasis as well as autonomous secretion of PTH.
**Prevalence**	Majority of primary hyperparathyroidism cases (80%).	Extremely rare, comprising approximately 1–5% of cases of primary hyperparathyroidism.
**Laboratory**	Hypercalcemia (usually >1.0 mg/dL above the upper normal level) and elevated PTH levels (or inappropriately normal levels).	Markedly elevated, often significantly higher than in adenoma. PTH levels up to 10 times the normal value and hypercalcemic crisis in up to 12% of patients (serum calcium > 14 mg/dL).
**Symptoms**	Hyperparathyroidism (nephrolithiasis, ostealgia, fractures on pathological bone, osteoporosis, fatigue, neuropsychiatric symptoms, polydipsia, polyuria, nausea, and vomiting).	Hyperparathyroidism + compressive symptoms of the upper airway and digestive tract. Systemic illness if metastases.
**Diagnosis**	Serum biochemical analysis (calcium, PTH) and imaging modalities (ultrasound, SPECT, or CT).	Confirmed through histopathological examination.
**Treatment**	Surgical excision of parathyroid adenoma.	En bloc resection of the primary tumor, ipsilateral thyroid lobe and isthmus + involved pre-thyroid muscles and central neck lymph node dissection; may necessitate adjunctive therapies (radiotherapy or chemotherapy).
**Prognosis**	Generally favorable with surgical intervention, with low rates of recurrence.	Prognosis is variable; generally poor, especially in cases with distant metastasis. Mortality secondary to refractory hypercalcemia.

**Table 3:** Genomic, epigenetic, proteomic, and inflammatory markers studied in PC.

	Marker	Characteristics	References
**Genomics**	**CDC73 gene (HRPT2)**	Tumor suppressor gene that encodes a parafibromin; main genetic alteration in sporadic PC.	[54,55]
	**PRUNE2 gene**	PRUNE2 protein: tumor suppressor activity by suppressing the activity of RhoA.	[55,56]
	**CCND1 gene**	Encodes cyclin D1, amplified in 71–90% of PC. Associated with cell proliferation.	[52,55]
	**MEN1 gene**	Mutations cause multiple endocrine neoplasia type 1. PC: 13 to 31%.	[55]
	**TERT gene**	Somatic mutations; associated to telomerase activity through the coding of its catalytic subunit.	[55]
	**ZEB1 gene**	EMT-TF.	[46,55]
**Epigenetics**	**miR-27a-5p**	Significant upregulation. Activation of the Wnt/β-catenin signaling pathway. AUC: 0.86.	[60]
	**miR-342-3p**	Significantly decreased in PC. AUC: 0.89.	[59,61]
	**HGS mRNA**	Upregulated. hepatocyte growth factor receptor-regulated tyrosine kinase substrate. EMT-related phenomena such as downregulation of E-cadherin.	[62]
	**miR-296**	Downregulated in PC. HGS mRNA is a direct target of miR-296, and miR296 undergoes significant downregulation during tumor progression.	[62]
	**profile [LINC00959, lnc-FLT3-2:2, lnc-FEZF2-9:2, and lncRP11-1035H13.3.1-2:1]**	AUC: 0.88, Sen: 81.8%, and Spe: 83.9%.	[66]
	**lncRNAs PVT1**	AUC: 0.871.	[67]
	**circRNA_0075005**	AUC: 0.77.	[58,65]
**Proteomics**	**UCH-L1 (or PGP9.5)**	Overexpressed; part of the deubiquitinase family and is associated with carcinogenesis pathways.	[55,68]
	**ANXA2**	Aggressiveness marker in gastrointestinal cancers. Calcium-mediated binding to membrane phospholipids.	[55,68]
**Inflammatory**	**Lymphocyte** **–** **monocyte ratio**	Decreased, independent predictor of PC if <4.85.	[69]

## Data Availability

All supporting data of this manuscript are available upon request to the corresponding authors.
